# Current Perspectives in Mesenchymal Stromal Cell Therapies for Airway Tissue Defects

**DOI:** 10.1155/2015/746392

**Published:** 2015-06-08

**Authors:** Francesco Petrella, Stefania Rizzo, Alessandro Borri, Monica Casiraghi, Lorenzo Spaggiari

**Affiliations:** ^1^Department of Thoracic Surgery, European Institute of Oncology, 20143 Milan, Italy; ^2^Department of Radiology, European Institute of Oncology, 20143 Milan, Italy; ^3^University of Milan School of Medicine, 20122 Milan, Italy

## Abstract

Lung cancer is the leading cause of cancer death and respiratory diseases are the third cause of death in industrialized countries; for this reason the airways and cardiopulmonary system have been the focus of extensive investigation, in particular of the new emerging branch of regenerative medicine. Mesenchymal stromal cells (MSCs) are a population of undifferentiated multipotent adult cells that naturally reside within the human body, which can differentiate into osteogenic, chondrogenic, and adipogenic lineages when cultured in specific inducing media. MSCs have the ability to migrate and engraft at sites of inflammation and injury in response to cytokines, chemokines, and growth factors at a wound site and they can exert local reparative effects through transdifferentiation and differentiation into specific cell types or via the paracrine secretion of soluble factors with anti-inflammatory and wound-healing activities. Experimental and clinical evidence exists regarding MSCs efficacy in airway defects restoration; although clinical MSCs use, in the daily practice, is not yet completely reached for airway diseases, we can argue that MSCs do not represent any more merely an experimental approach to airway tissue defects restoration but they can be considered as a “salvage” therapeutic tool in very selected patients and diseases.

## 1. Introduction

Lung cancer is the leading cause of cancer death and respiratory diseases are the third cause of death in industrialized countries; for this reason the airways and cardiopulmonary system have been the focus of extensive investigation, in particular of the new emerging branch of regenerative medicine.

Exposure to environmental insults damages the cells of the lung; thus the lung has a wound-healing capacity that promotes tissue regeneration and/or restoration by proliferation and differentiation of stem and progenitor cells.

The reparative attitude of adult human tissues falls along an injury response spectrum: at one end there are tissues with a constitutively high rate of cell turnover and a well-delineated stem/progenitor cell hierarchy, like epidermis, intestine, and hematopoietic system; at the other end there are organs containing few stem cells and cannot repair efficiently, resulting in scarring after injury, like heart and brain; in between these two extremes are tissues that have a low steady state cell turnover and can react after injury to replace damaged cells, like lung, liver, and pancreas.

Large airway defects and tracheobronchial dehiscence following curative lung resection present a major problem for clinicians because no effective methods of treatment are available.

Postresectional bronchopleural fistula (BPF) is a pathological connection between the airway (bronchus) and the pleural space that may develop after lung resection and may be caused by incomplete bronchial closure, impediment of bronchial stump wound healing, or stump destruction by residual neoplastic tissue.

The clinical effect of impaired bronchial stump healing after anatomic lung resection may culminate in a life-threatening septic and ventilatory catastrophe. For many patients with empyema, the presence or absence of a fistula makes the difference between recovery, chronicity, and death.

Mesenchymal stromal cell therapy may represent a therapeutic option for this unsolved problem and for several other diseases of the respiratory tract, like COPD and ARDS.

## 2. Mesenchymal Stromal Cells

Mesenchymal stromal cells (MSCs) are a population of undifferentiated multipotent adult cells that naturally reside within the human body and are generally defined as plastic-adherent, fibroblast-like cells possessing extensive self-renewal properties and potential to differentiate* in vivo* and* in vitro* into a variety of mesenchymal lineage cells [1]; they can differentiate into osteogenic, chondrogenic, and adipogenic lineages when cultured in specific inducing media [2].

MSCs are described as Major Histocompatibility Complex II (MHC II) negative cells, lacking costimulatory molecules such as CD40, CD80, and CD86, thus having an immune phenotype (MHC II^−^, CD40^−^, and CD86^−^) allowing evading the host immune system, thus permitting allogenic transplantation without immunosuppression [3].

The immunomodulatory and anti-inflammatory effect of MSCs have been extensively studied and used in the gastrointestinal tract, like in inflammatory bowel disease and graft-versus-host disease [4, 5]; it has been recently demonstrated that MSCs derived from Crohn's patients deploy indoleamine 2,3-dioxygenase-mediated immune suppression [6].

Once implanted, MSCs are able to interact with the surrounding microenvironment, promoting tissue healing and regeneration, renewing biologic function by supportive and trophic functions based on cross talk with other cells present within diseased tissues [7]. MSCs have been shown to exert profound anti-inflammatory and immunomodulatory effects on almost all the cells of the innate and adaptative immune system by a variety of mechanisms, notably cytokine and chemokine secretion, like Interleukin-10 (IL-10), Interleukin 6 (IL-6), Transforming Growth Factor Beta (TGFB), Vascular Endothelial Growth Factor (VEGF), Intercellular Adhesion Molecules (ICAMs), and Prostaglandin E2 (PG E2) [8].

After their initial discovery in bone marrow, MSCs were isolated and characterized from a wide variety of other adult and fetal tissue, including adipose tissue [9], umbilical cord [10], dental pulp [11], tendon [12], thymus, spleen [13], cornea [14], liver [15], brain [16], periosteum [17], placenta [18], and synovial and amniotic fluids [19].

MSCs isolated from these different tissues are different, although no significant difference in the profiles of secreted cytokines by different type of MSCs has been described; some quantitative differences in the cytokine secretions by adipose tissue-derived MSCs (AT-MSCs) and bone marrow-derived MSC (BM-MSC) have been reported [20].

Besides the trilineage differentiation potential into osteoblasts, adipocytes, and chondroblasts in* in vitro* culture with specific stimuli, experimental data have demonstrated that MSCs can also differentiate into other mesodermal lineages, such as skeletal myocytes, cardiomyocytes, tenocytes, and endothelial cells; moreover MSCs have the capacity to differentiate into types of cells of endodermal and ectodermal lineages, including hepatocytes, neuronal cells with neuron-like functions, insulin-producing cells, photoreceptor cells, renal tubular epithelial cells, and epidermal and sebaceous duct cells [8]. MSCs have the ability to migrate and engraft at sites of inflammation and injury in response to cytokines, chemokines, and growth factors [21] at a wound site and they can exert local reparative effects through transdifferentiation into tissue-specific cell types or via the paracrine secretion of soluble factors with anti-inflammatory and wound-healing activities [22].

## 3. Mesenchymal Stromal Cells in Respiratory System

The lung is a highly quiescent tissue, previously thought to have limited reparative capacity and a susceptibility to scarring [23]; we now know that the lung has a remarkable reparative capacity, when needed, in response to specific stimuli and injuries [24].

The tissues of the lung may be categorized as having facultative progenitor cell populations that can be induced to proliferate in response to injury as well as differentiate into one or more cell types [24]; given the complexity of the respiratory system, a single lung stem cell generating all of the various lineages within the lung is difficult to conceive: the two most likely hypotheses are that the lung could respond to injury and stress (a) by activating stem cell populations or (b) by reentering the cell cycle to repopulate lost cells [24].

During lung embryonic development, rapid proliferation and differentiation are the rule rather than the exception; on the contrary, in the adult lung during postnatal life, it is not clear whether any lung cells of comparably expansive proliferative potential or differentiation repertoire still remain active, and so we refer to these developing cells as progenitors rather than stem cells, as their self-renewal capacity may be transient [24].

We can identify, within the respiratory system, at least four different districts in which different stem cell candidates may be considered: (1) trachea and proximal bronchi, (2) distal airway system, (3) alveolar compartment, and (4) bronchoalveolar duct junction.

The trachea and main stem bronchi are lined with pseudostratified epithelium composed of basal and luminal cells; subsets of basal cells, both in mice and in humans, have extensive proliferative potential, self-renewal capacity, and the ability to differentiate into basal, secretory, and ciliated lung epithelial cells* in vivo* [25]; considering that basal cells have no other known function in the lung, this supports the concept that basal cells can function as tissue-specific stem cells of the airway epithelium, although little is known about basal cell self-renewal and differentiation and whether it involves asymmetric cell division as do other stem cells [24].

In the distal airway the bronchiolar epithelium is quiescent until injured; a subset of secretory cells, named variant club cells, show proliferation potential in response to injury but it is still unclear if they go through a process of dedifferentiation to reenter the cell cycle and then differentiate again after expansion [26]; these cells can be found adjacent to the neuroendocrine bodies or at the bronchoalveolar duct junction, confirming the hypothesis of the existence of microenvironmental progenitor cell niches in the airways [27].

The type II alveolar epithelial cells are considered the best candidate for progenitor cells of the adult lung alveolus [28] during the late development, in fact, or after various postnatal alveolar injuries; some type II alveolar epithelial cells can proliferate, self-renew, and form alveolar epithelial cells type I [29] presenting self-renewal signals like epidermal growth factor receptor (EGFR) [30].

At the transition from the bronchiolar region to the alveolar region of the lung there is the bronchoalveolar duct junction, where some variant club cells possess airway epithelial regenerative potential after induced lung injury [24], defined as bronchoalveolar stem cells (BASC); however the existence of BASC* in vivo* has been contested [31] so further studies are required to consider BASC as true stem cell lineage existing in a unique niche between the airways and alveoli [24].

## 4. Mesenchymal Stromal Cells for Lung and Airway Diseases

The main function of stem/progenitor cells for the airway epithelium is epithelial homeostasis and the repair of defects in the airway wall [32].

Stem/progenitor cells can be used to repair defects in the airway wall, resulting from tumors, trauma, tissue reactions following long-time intubations, or diseases that are associated with epithelial damage [33].

In many airways diseases such as asthma, chronic obstructive pulmonary diseases, obliterative bronchiolitis, and cystic fibrosis, the airway epithelium is damaged and subsequently repaired and remodeled [34].

Reconstruction of tracheobronchial defects requires in the first place the availability of airway epithelial cells and the presence of fibroblasts or fibroblast-derived substances [33].

The fact that fibroblasts have positive effects on airway epithelial cell growth emphasizes the fact that the airway is not a simple structure and that epithelial-mesenchymal interactions are important [33].

**Figure 1:**
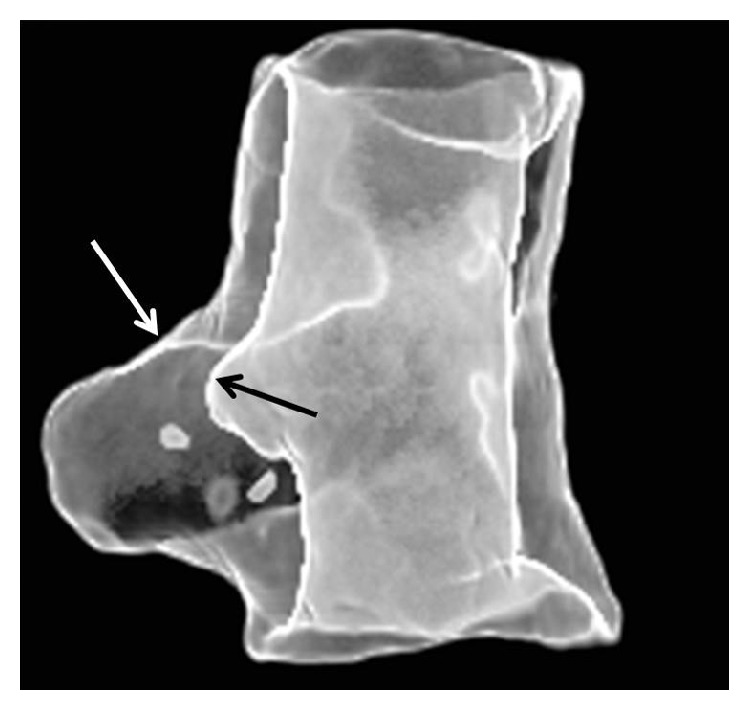
Computed tomography shows the internal (black arrow) and external (white arrow) surfaces of the regenerated bronchial wall, in a large animal model, of a right bronchopleural fistula, demonstrating abundant peribronchial tissue occluding the bronchial stump.

Considering the catastrophic consequences that airway tissue defects may have after lung resection, culminating in a pathological communication between the airways and the pleural space called “bronchopleural fistula” (BPF), we proposed, on an animal model, an autologous bone marrow-derived mesenchymal stem cells (BMMSC) transplantation: it allowed bronchial stump healing by extraluminal fibroblast proliferation and collagenous matrix development [35] (Figure 1).

**Figure 2:**
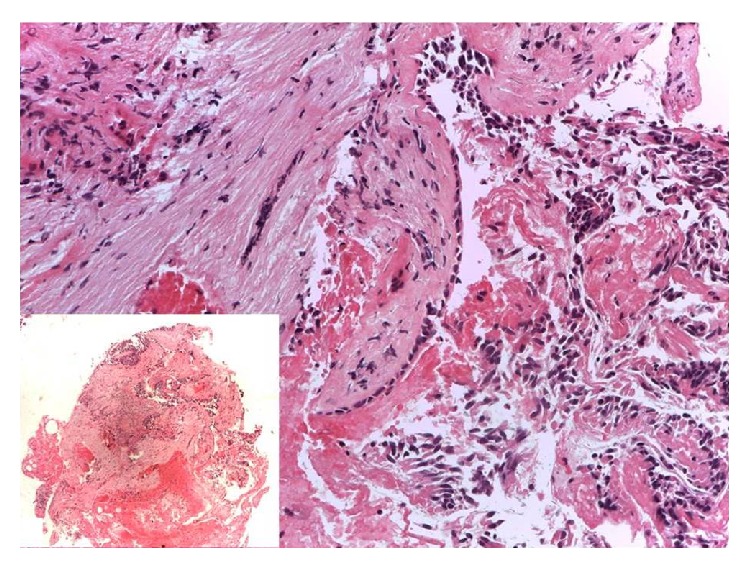
The specimen of bronchial mucosa obtained by human bronchoscopic biopsy 60 days after stem cell implantation showed a discrete coarctation induced by sample taking (left lower box); however it was possible to appreciate hyperplastic respiratory epithelium lying on a diffusely fibrotic lamina propria. Bands of smooth muscle fibers were reduced and replaced by fibroblasts.

**Figure 3:**
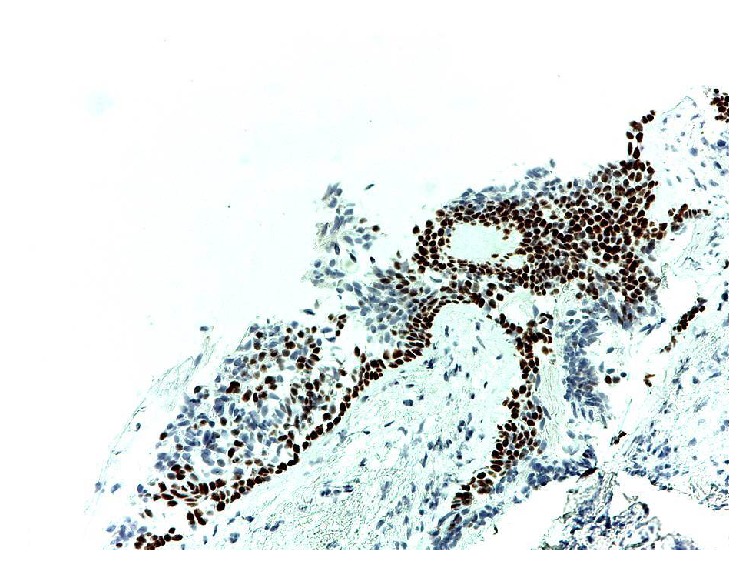
Immunocytochemical stain for p40 showing a well-defined layer of basal cells and basal cell hyperplasia consistent with repair.

Encouraged by experimental bronchial wall restoration in large animals and by functional human organ replacement elsewhere [36], we undertook autologous BMMSC bronchoscopic transplantation to treat a patient who developed BPF after right extrapleural pneumonectomy for malignant mesothelioma [37]. The bronchoscopic transplantation of bone marrow-derived mesenchymal stem cells in our patient appeared to help close this small-caliber postresectional bronchopleural fistula, further boosting regenerative medicine approach for airway diseases (Figures 2 and 3).

There are a number of ongoing clinical trials addressing the feasibility and safety of MSCs treatment for airway diseases, focusing on the role of human MSCs for the treatment of subjects with moderate to severe chronic obstructive pulmonary disease [38, 39].

## 5. Mesenchymal Stromal Cells Imaging

The serial visualization and tracking of transplanted mesenchymal stem cells, including the assessment of their presence at the site of injection and their possible migration or retention in other sites, are still issues to be resolved.

Optical methods, mainly based on retroviral vectors to express fluorescent proteins, allow visualization of cells that homed in different organs only after sacrifice of the animal, as the tissue penetrability of fluorescence is limited [40].

Therefore, other techniques, able to track injected MSCs* in vivo*, such as positron emission tomography (PET), single-photon emission CT (SPECT), and magnetic resonance (MR), have being employed.

PET imaging can be performed using direct and indirect labeling approaches. Direct approaches are based on labeling stem cells with radioactive compounds such as [^18^F]-fluorodeoxyglucose. In proliferating cells the radioactive compound is distributed to daughter cells; therefore the signal measured in the cells will shrink due to proliferation [41, 42] and will be visible for a short period of time, as the tracer decays over 109 minutes [43]. Moreover, the direct labeling approach is associated with high efflux and low intracellular stability. Indirect approaches rely on the activation of a tracer dye by a protein, such as herpes simplex virus type 1 thymidine kinase (HSV1-TK), transduced by a recombinant viral vector into the cells [44, 45]. After application, the tracer will be phosphorylated through HSV-TK leading to metabolic trapping in the recombinant cells. Studies demonstrated the feasibility of this technique for monitoring cell fate* in vivo*, after myocardial administration [46] and in healing after injuries [47].

SPECT uses the radioactive decay of radionuclides and gamma rays to provide 3D information on cell location using tomographic reconstruction. Most usable and FDA-approved SPECT isotopes are short-lived (e.g., Tc-99 m (360 minutes), Ga-67 (4320 minutes), In-111 (4020 minutes), and I-123 (780 minutes)) [48]. SPECT can also be combined with PET and CT imaging and has been successful in imaging labeled human MSCs (hMSCs), in animal models, although the effects of emission from a tracer dye can be toxic and may interfere with hMSC functions [49, 50].

Both PET and SPECT have the ability to provide functional myocardial data and therefore have been studied mostly in the context of myocardial ischemia [51].

Thanks to its capacity for high spatial resolution (ranging from 50 *μ*m in animals up to 300 *μ*m in whole body clinical scanners), magnetic resonance (MR) has been considered an excellent method for tracking cells* in vivo*. Cells can be labeled either with positive contrast agents, used in T1-weighted MRI such as gadolinium, or with negative contrast agents, such as superparamagnetic iron oxide (SPIO) and ultrasmall superparamagnetic iron oxide (USPIO) particles [40, 52], which are highly sensitive and have a dominant effect on the T2/T2∗ relaxation times. Most cell tracking studies have used SPIO and USPIO to label stem cells for detection with MR, due to the pronounced signal change that even small amounts of these contrast media can create (owing to the so-called blooming artifact). This allows the detection of even very small numbers of labeled cells [53, 54]. With such agents, MRI has been shown to allow the location of iron-labeled cells to be noninvasively monitored* in vivo* over several weeks [55–57]. However, drawbacks of labeling by ferromagnetic nanoparticles include that other endogenous sources of signal loss may appear in images that are sensitive to iron (e.g., due to blood, hemosiderin, bone, and air) making it difficult to unambiguously identify regions containing labeled cells [58, 59]. Another drawback is that the iron oxide particles may be retained in a tissue, even if the grafted (stem) cell dies, hence leading to false positive signals [48]. Moreover, most FDA-approved SPIOs have now been discontinued from the market, so moving to clinics with SPIO-labeled cells will be difficult in the near future.

Emerging MR imaging techniques are under evaluation, such as imaging based on perfluorocarbon formulations, whose advantage is the high specificity due to the virtual absence of fluor from the body [59]. The fluorine signal can be accurately quantified from the MR images by comparing the ^19^F signal in the tissue of interest to an external reference containing a known amount of fluorine atoms. The principal drawback of the fluor-based MR imaging is its relatively low sensitivity when compared to cellular imaging with iron nanoparticles.

It is therefore clear that the choice of an imaging technique will rely on the efficacy, toxicity, and resolution considered the best for the specific research setting under evaluation.

## 6. Conclusion

**Figure 4:**
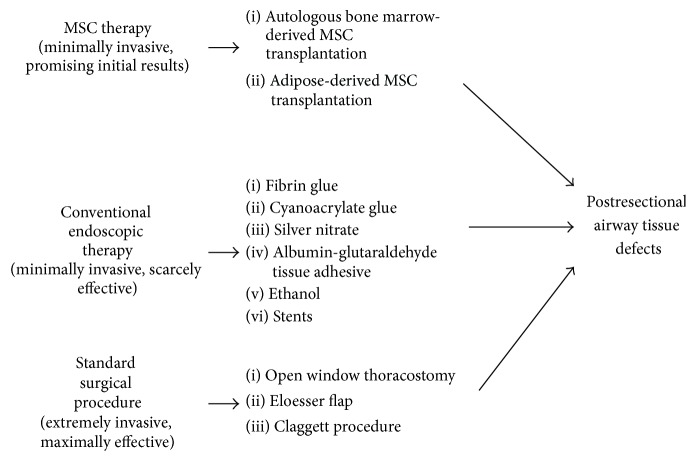
An algorithm showing standard, endoscopic, and MSC based therapies for postresectional airway tissue defects.

Experimental and clinical evidence exists regarding MSC efficacy in airway defects restoration [35, 37, 60]; although clinical MSC use, in the daily practice, is not yet completely reached for airway diseases, we can argue that MSCs do not represent any more merely an experimental approach to airway tissue defects restoration but they can be considered as a “salvage” therapeutic tool in very selected patients and diseases (Figure 4).

Although some concerns have been expressed with regard to MSCs potential of neoplasm development in cancer patients [61], no clear evidence exists in particular in case of MSC injection in tissue free of cancer and without “*in situ*” neoplasms [62].

Interesting clinical and experimental results have been obtained by MSCs therapy for large bone defects [63, 64].

Encouraging results have been reported for the treatment of acute respiratory distress syndrome with allogeneic adipose-derived mesenchymal stem cells in a randomized, placebo-controlled pilot study, showing that administration of allogenic adipose-derived MSCs appears to be safe and feasible in the treatment of ARDS [65] as well as in a phase I clinical trial disclosing that a single intravenous infusion of allogeneic, bone marrow-derived human MSCs was well tolerated in nine patients with moderate to severe ARDS [66].
